# Evaluation of Early versus Delayed Laparoscopic Cholecystectomy in Acute Cholecystitis

**DOI:** 10.1155/2015/349801

**Published:** 2015-02-03

**Authors:** Rati Agrawal, K. C. Sood, Bhupender Agarwal

**Affiliations:** DNB (General Surgery), Department of General Surgery, Maharaja Agrasen Hospital (MAH), New Delhi 110026, India

## Abstract

*Background*. The role of early laparoscopic cholecystectomy for acute cholecystitis with cholelithiasis is not yet established. The aim of our prospective randomized study was to evaluate the safety and feasibility of early LC for acute cholecystitis and to compare the results with delayed LC. *Methods*. Between March 2007 to December 2008, 50 patients with diagnosis of acute cholecystitis were assigned randomly to early group, *n* = 25 (LC within 24 hrs of admission), and delayed group, *n* = 25 (initial conservative treatment followed by delayed LC, 6–8 weeks later). *Results*. We found in our study that the conversion rate in early LC and delayed LC was 16% and 8%, respectively, Operation time for early LC was 69.4 min versus 66.4 min for delayed LC, postoperative complications for early LC were 24% versus 8% for delayed LC, and blood loss was 159.6 mL early group versus 146.8 mL for delayed group. However early LC had significantly shorter hospital stay (4.1 days versus 8.6 days). *Conclusions*. Early LC for acute cholecystitis with cholelithiasis is safe and feasible, offering the additional benefit of shorter hospital stay. It should be offered to the patients with acute cholecystitis, provided that the surgery is performed within 96 hrs of acute symptoms by an experienced surgeon.

## 1. Introduction

For the management of acute cholecystitis with cholelithiasis the appropriate timing for laparoscopic cholecystectomy remains controversial [1]. Two approaches are available for the treatment of acute cholecystitis; the first approach is early (within 7 days of onset of symptoms) [2–5] laparoscopic cholecystectomy (LC) as definitive treatment after establishing diagnosis and surgical fitness of the patient in the same hospital admission. The second approach is conservative treatment which is successful in about 90% of the cases and then delayed cholecystectomy is performed in the second hospital admission after an interval of 6–12 weeks [6]. The choice of approach depends upon hospital infrastructure, surgical expertise, and patient's condition.

In the presence of acute inflammation, LC becomes more challenging and difficult because of edema, exudate, adhesions with adjoining structures, distension of gallbladder, friability of tissues, unclear and distorted ductal and vascular anatomy [7], hypervascularity, congestion, and dissemination of infection. These risk factors predispose for suboptimal outcome and high conversion rate to open cholecystectomy. As a result, the patient is deprived of potential benefits of LC which is now a “gold standard” for the management of symptomatic gallbladder stones [8].

Early open cholecystectomy had been established as the preferred treatment of acute cholecystitis to reduce morbidity, mortality, and total hospital stay [9]; however, with the advent of LC, the benefits of early surgery have been the subject of some contention [10]. Initial reports suggested that early LC for acute cholecystitis was associated with increased complication rates, prolonged operation time, and increased conversion rates (5%–35%) [1, 11–13]. As a consequence, initial conservative management with subsequent delayed or elective LC became accepted practice [9, 14, 15].

Delayed cholecystectomy potentially increases the chance of further gallstone-related complications [4] during the waiting interval and thus additional hospital admission. Recent evaluation has indicated early LC to be safe option in acute cholecystitis, although conversion to open cholecystectomy rates may be higher [11–13, 16].

## 2. Aims and Objectives

The aims and objectives of this study are as follows:

1. to evaluate the results of early laparoscopic cholecystectomy in patients with acute cholecystitis with cholelithiasis in our setup with attention to clinical outcome,
2. to compare the results of early with the delayed laparoscopic cholecystectomy for the treatment of acute cholecystitis with cholelithiasis.

## 3. Material and Methods

This prospective randomized study was undertaken in the Department of surgery at Maharaja Agrasen Hospital (MAH), New Delhi, between March 2007 and December 2008. Total of 50 patients were included in the study irrespective of their age and sex. Patients coming to the Emergency/Out Patient Department of MAH within 72 hrs of acute symptoms were diagnosed as a case of acute cholecystitis on the basis of clinical, laboratory (acute upper abdominal pain, right hypochondrial tenderness and/or guarding, fever >37.5°C and/or, white blood cell count greater than 10 × 10^9^/L), and ultrasonographic criteria (thickened > 4 mm and edematous gallbladder (GB), distended gallbladder, positive sonographic Murphy's sign, pericholecystic fluid, and gallstones). According to* Tokyo guidelines 2013*, all our patients belong to* severity grade I (mild)*. Magnatic tesonance cholangiopancreaticography (MRCP) was done in equivocal cases and findings correspond to the T2 single shot coronal image showing distended gallbladder, circumferential gallbladder wall thickening, and increased hyperintensity and T2 STIR axial image showing a distended gallbladder, cholelithiasis, and pericholecystic T2 hyperintense inflammation. Patients with acute symptoms present for >96 hrs prior to admission, previous upper abdominal surgery, patients unfit for general anaesthesia, coexisting common bile duct (CBD) stones as suggested by history of jaundice or fever with chills, icterus, raised alkaline phosphatase, or ultrasonographic evidence of CBD calculus, coexistent acute cholangitis, or pancreatitis were excluded from the study.

### 3.1. Workup of Patients

Eligible patients were talked to about the 2 options of treatment (early and delayed laparoscopic cholecystectomy) and informed consent was obtained. Patients were then randomized into two groups, “early” and “delayed” groups. Randomization was accomplished according to the patient's choice of treatment for some socioeconomic reasons. We used both the interview (for history and analgesic requirement) and the observation check list (for lab results, radiological and operative findings). In the early group, laparoscopic cholecystectomy was performed within 24 hours of randomization, that is, within 96 hours of acute symptoms, whereas in the delayed group conservative management with intravenous fluids and antibiotics was done. Patients who responded to the conservative management underwent an elective laparoscopic cholecystectomy 6–8 weeks after the acute episode.

### 3.2. Conduct of the Operation

The nature of the surgery, chance of conversion to open cholecystectomy, and the benefits likely to be achieved from LC were explained to the patients and the relatives in detail. After obtaining an informed written consent and randomization, patients were taken up for the surgery. Laparoscopic cholecystectomy, whether early or delayed, was performed by a consultant surgeon. The surgery was performed under general anaesthesia using endotracheal intubation in supine position.

Nasogastric tube was inserted to decompress the stomach. Pneumoperitoneum was created by blind puncture with Veress needle through a supraumblical incision. Confirmation of the intraperitoneal location of the needle tip is made by the saline drop test; once the needle is confirmed to be in the right position, the peritoneal cavity is insufflated, using carbon dioxide. To prevent problems of venous return, the pressure should never exceed 15 mm Hg. Four laparoscopic ports were made. The epigastric 10 mm port was for dissection or the suction and retrieval of specimen. Five mm port was made for telescope Three 5 mm ports were placed one in supraumblical region, one in right upper quadrant, and another in right flank at level of umbilicus were used for grasping forceps. If necessary, fifth port was added to improve exposure. Adhesions if present were cleared and gallbladder exposure was first undertaken; then the positions of gallbladder, the first part of the duodenum, common bile duct, Calots' triangle, and porta hepatis were ascertained. The gallbladder, if distended, was decompressed through suction needle to allow better grasping.

The gallbladder is held above the liver and the omentum and duodenum retracted caudally to define the neck of GB, Calot's triangle, and the common bile duct or lateral margin of the portal triad. Separation of the tissues to isolate structures in Calot's triangle by atraumatic manner is especially useful in AC.

The dissection is always started at the junction between the cystic duct and gallbladder at the inferior margin and carried out upwards close to the gallbladder neck on its posterior aspect with complimentary anterior dissection in Calot's triangle. Posterior window is created by separating the neck and part of body of the gallbladder all around. Next dissection is downward from the junction of gallbladder neck and the cystic duct, to define the cystic duct and the cystic artery. At the completion of this stage a critical view of safety is taken to ascertain that the two structures; that is, cystic duct and cystic artery are joining the gallbladder clearly. Dissection begins in the triangle of Calot taking small bands and strands of tissue. The cystic pedicle was dissected with curved dissector in order to isolate separately the cystic duct and artery. Both elements were then clipped and divided [7]. Gallbladder was dissected off its bed by to and fro retraction with a monopolar cautery hook. At the completion of the procedure, the gallbladder was placed into a retrieval bag if needed and extracted through the epigastric port, which was enlarged if necessary. Hemostasis was achieved in the gallbladder bed and after a thorough saline lavage, a suction drain was left in place if clinically indicated and the ports closed. When required, the conversion to open procedure was performed through a right subcostal incision.

### 3.3. Postoperative Assessment

Postoperatively, the patients were allowed oral intake 6–12 hrs after surgery if they had no nausea or vomiting. Pain relief was obtained by intramuscular diclofenac injection, which was changed to oral once patient was allowed orally. The severity of pain was documented by daily pain scoring using a visual analog scale (VAS 0: no pain; VAS 10: intolerable pain) for 3 days.

### 3.4. Study Parameters

Data was collected and entered in a predesigned proforma(Annexure-I) which included patient's demographics, timing of operation, operative findings, operative time, intra- or postoperative complications, and the length of hospital stay.

### 3.5. Statistical Analysis

Data was statistically analysed by using student *t*-test, Fisher's exact test, and Wilcoxon rank-sum (Mann-whitney) test. A *P* value < 0.05 was considered significant.

## 4. Results

During the study period, a total of 50 patients with acute cholecystitis were included in the study. They were randomized in early and delayed groups with 25 patients in each group. The two groups were comparable in terms of age and sex, as well as clinical, laboratory parameters, and ultrasonographic findings (Table 1). No patient in delayed group required urgent surgery for failure of conservative treatment or recurrent symptoms during waiting period. Delayed laparoscopic cholecystectomy was performed at a mean interval of 47.32 days (6.76 weeks).

*USG Finding on Initial Advice.* See Table 1.

*Modification of Technique.* The various modifications in the techniques used in our studies are shown in Table 2.

*Operation Time and Blood Loss.* More modifications in the operative technique (Table 2) were required in early group than in the delayed group. The mean operating time was 60 min (range: 35–150 min) in early group and 60 min (range: 45–100 min) in delayed group, which is statistically not significant (*P* = 0.8004). The average blood loss in early LC was 159.6 mL (±58.1) and in delayed LC was 146.8 mL (±10.5). The difference in blood loss was statistically not significant (*P* = 0.418), and no patient required blood transfusion postoperatively.

*Conversion to Open Cholecystectomy.* In early group 21 cases were completed successfully by laparoscopy and 4 were converted to open cholecystectomy. Conversion rate was 16%. In delayed group 23 cases were successfully completed by laparoscopy and 2 were converted to open cholecystectomy. Conversion rate was 8%. The reasons for conversion in early group were as follows:

1. unclear and distorted anatomy of ductal and vascular structures in Calot's triangle due to dense adhesions, edema, and exudates,
2. tearing of GB at Hartmann's pouch because of friable tissue,
3. bile leakage from cystic duct with suspicion of injury to common bile duct.

In our study, as shown in Table 3, conversion to open cholecystectomy was done in 4 patients due to dense adhesions; in two of them, on opening fundus, the first method was employed and anatomy was still unclear, so gallbladder was transected at the lower part of gallbladder neck, stones were removed, and the gallbladder wall was repaired. Patients recovered uneventfully. In the other two, structures could be defined clearly on opening and standard cholecystectomy was done.

In delayed group, only 2 patients were converted to open cholecystectomy. These patients had several previous episodes of acute cholecystitis and biliary pain and could be operated on earlier because of socioeconomic reasons. There were thickened dense adhesions and anatomy was unclear so conversion to open was done.

- One patient with bile leak had tear in the cystic duct and CBD was intact.
- One patient with tearing of gallbladder neck was treated with standard open cholecystectomy.

*Complications.* There was no death in any of these two groups. The overall complication rate was 32% (8 of 25) in early group and 8% (2 of 25) in the delayed group. There was no major bile duct injury in any patient. In delayed group only one patient had chest infection and was managed with chest physiotherapy and intravenous antibiotics. There was high rate of wound infection in early group. Different complications are shown in Table 4.

*Postoperative Analgesia.* The average VAS score of postoperative analgesia was 2 in early group and 2 in delayed group, which was not statistically significant (*P* = 0.673).

*Length of Hospital Stay (HS).* The mean total HS was 4.16 (3–6 days) in the early and 8.6 (3–13 days). The difference in total HS between two groups was statistically significant (*P* = 0.0001), as shown in Table 5.

The overall comparison of the patients in the early and delayed groups is shown in Table 6.

In our study the parameters measured are depicted in Table 6. There is no statistically significant difference in both groups except the total hospital stay which is less in early group being 4.16 ± 1.21 days in comparison to 8.6 ± 2.04 in delayed group.

## 5. Discussion

Laparoscopic cholecystectomy was started in 1987 and in few years became “gold standard” for the treatment of symptomatic cholelithiasis and was also used for acute cholecystitis as more experience was gained in the technique. However, the application of LC in the setting of acute cholecystitis is still controversial. In early years of laparoscopic surgery, acute cholecystitis was considered a relative contraindication to LC [4, 17]. However, some recent reports [1–6, 8, 18–26] have suggested that LC is feasible and safe procedure for acute cholecystitis also, although the complications and conversion rates are variable. However, more studies are required for conclusive results.

We, therefore, undertook a prospective randomized trial comparing early versus delayed LC for acute cholecystitis and also to evaluate feasibility and safety in our set up. The patients' population was well-matched in both groups and there was no significant difference in age, biochemical parameters, and radiological findings between 2 groups.

The mean operation time in our study was 69.4 min in early group and 66.4 min in delayed group. The difference was not statistically significant. This is in contrast to the reports from other trials which showed a significant difference in operative time between two groups.

There was no significant difference in blood loss between the two groups; the mean blood loss in early group was 159.6 mL and 146.8 mL in delayed group. Although there are not several studies which compared the difference in the blood loss, more blood loss in early group is due to highly vascular adhesions around inflammatory GB and oozing from inflammatory GB bed; however, no patient in the study required blood transfusion.

More surgeons agree that in acute cholecystitis timing of cholecystectomy is an important factor in determining outcome. Ideally the surgery should be performed as soon after admission as possible. Although operation within golden 72 hrs from the onset of symptoms has been suggested, such an early surgery is not always possible in clinical practice because of logistic difficulties in operating such patients on an emergency basis. We perform the surgery for the patient in early group in the next available OT (elective list). >90% of our patients had surgery within 24 hrs of admission.

The technical difference of LC is related to operative findings during early surgery. A diseased GB containing infected bile is commonly seen in acute cholecystitis. We believe that several technical key points must be kept in mind while performing lap surgery for acute cholecystitis. For a good exposure of Calot's triangle, an additional port can be useful. Decompression of GB allows better grasping of GB by grasper. If available, ultrasonic dissector and coagulator should be used for adhesionolysis. In difficult cases with dense adhesions between the GB neck and porta hepatis, a partial cholecystectomy can be done leaving in place Hartmann's pouch and the cystic duct after confirming the absence of distal residual structures. The other technical rules are liberal use of subhepatic drain and a retrieval bag to remove spilled stones and perforated GB. In our study decompression of GB was required in 64% cases in early group and 12% cases in delayed group. Retrieval bag was used in 16% cases of early group and in 4% cases in delayed group. Subhepatic drain was placed in 28% pts of early group and 8% patients of delayed group.

Three questions have to be answered regarding LC in the setting of acute cholecystitis.

*(1) Is Lap Chole for Acute Cholecystitis Safe?* The overall complication rate in this study was 20%. It is comparable to that reported in other studies. Postoperative complication rate in early group was 24% and in delayed group was 8%. This difference was statistically not significant and was also reported the same by Johansson et al. [5] (18% versus 8%) and Kolla et al. [6] (20% versus 15%). Study by Lai et al. [17] showed no difference in the complication rate between the two groups (9% versus 8%).

However, another prospective controlled study by Lo et al. [4] (29%) and González-Rodríguez et al. [25] (17.7%) had shown significantly higher complication in the delayed group than the early group.

The problem of biliary tract injury is the major concern in the routine use of the laparoscopic approach for acute cholecystitis. There was no mortality nor the major bile duct injuries in our study as were reported by Kum et al. [27] (5.5%) and Al-Hajjar et al. [28] (0.9%). Only the minor complications were more in early LC. These results suggest that the early LC is a safe procedure.

*(2) Is LC for AC Feasible?* In this study conversion rate in early group is more than the delayed group. It has been shown by many studies that LC for AC is feasible with conversion rate ranging from 6 to 35% [12, 13]. Although in our study conversion rate in early group is 16% which seems to be high, it reflects our safety concerns for the method and we feel that, with experience of these cases, the conversion rate will be lower in subsequent cases. The conversion rates in most of the studies lie in the acceptable range and are comparable to our study. So the procedure is feasible though the conversion rate in delayed cases is lower (8%) which has also been reported by various authors.

*(3) Is Lap Chole for Acute Cholecystitis Beneficial to the Patients?* The laparpscopic cholecystectomy for acute cholecystitis is definitely beneficial to the patient because of significantly shorter total hospital stay. The total hospital stay was significantly less for the early group (4.1 days) than the delayed group (8.6 days). Our study agrees with many other studies which also showed a significant difference in the hospital stay between both groups. There is socioeconomic advantage as well as prevention of recurrent attacks and complications during waiting period.

To conclude both early and delayed laparoscopic cholecystectomies are feasible and safe in acute cholecystitis; however, delayed lap chole is associated with lower conversion rate as compared to early LC; early cholecystectomy offers definitive treatment at the initial admission and avoids the problem of failed conservative management and recurrent symptoms which required emergency surgery. Furthermore, early LC is associated with a shorter total hospital stay as compared to delayed LC, which is a major economic benefit to the health care system especially in our country.

## Figures and Tables

**Table 1:** USG findings in early and delayed group.

Group	Early (*n* = 25)	Delayed (*n* = 25)	*P* value
Thickened GB	13 (52%)	21 (84%)	0.032
Distended GB	23 (92%)	21 (84%)	0.667
Gall stones	25 (100%)	25 (100%)	0.463
Murphy's sign	14 (56%)	14 (56%)	0.999
Pericholecystic fluid	6 (24%)	5 (20%)	0.999

*P* value < 0.05 is statistically significant.

Used Fisher's exact test.

**Table 2:** Various modifications of technique in early and delayed Group.

Technique	Early (*n* = 25)		Delayed (*n* = 25)		*P* value
	Frequency	Percentage	Frequency	Percentage	
GB decompression	16	64	3	12	0
Retrieval bag	4	16	1	4	0.349
Sub hepatic drain	7	28	2	8	0.138
Use of 5th port	1	4	0	0	0.999
Epigastric port enlargement	2	8	2	8	0.999

*P* value < 0.05 is statistically significant.

Used Fisher's exact test.

**Table 3:** Comparison of conversion to open cholecystectomy in early and delayed groups.

Procedure	Early (*n* = 25)	Delayed (*n* = 25)	*P* value
Successful LC	21	23	
Conversion to OC	4	2	
Conversion rate	16%	8%	0.667

*P* value < 0.05 is statistically significant.

Used Fisher's exact test.

**Table 4:** Comparison of operative complications in early and delayed groups.

Complications	Early (*n* = 25)		Delayed (*n* = 25)		*P* value
	Frequency	Percentage	Frequency	Percentage	
Intraoperative					0.353
Bile leak	1	4	0	0	
Perforation	1	4	0	0	
Postoperative					0.084
Chest infection	0	0	1	4	
Wound infection	6	24	1	4	

Total complications	8	32	2	8	

*P* value < 0.05 is statistically significant.

Used Fisher's exact test.

**Table 5:** Comparison of length of hospital stay in early and delayed group.

Hospital stay	Early (*n* = 25)		Delayed (*n* = 25)		*P* value
	Mean	SD	Mean	SD	
Postoperative HS	3	1	3.2	0.95	0.473

Total HS	4.16	1.21	8.6	2.04	0.0001

*P* value < 0.05 is statistically significant.

Used Wilcoxon rank-sum (Mann-Whitney) test.

**Table 6:** Overall comparison of early and delayed LC.

Groups	Early	Delayed	*P* value
Age	47.28 ± 14.57	50.96 ± 17.05	0.416
Sex	8 : 17	8 : 17	0.999
Duration of symptoms	35.44 ± 23.03	36.8 ± 21.30	0.209
TLC	10800 (6500–23200)	11200 (6600–18100)	0.341
Total bilirubin	0.76 (0.5–1.03)	1.8 (0.7–2.6)	0.05
SGOT	26 (15–94)	68 (14–99)	0.054
SGPT	28 (12–55)	97 (12–92)	0.095
Thickened GB	13 (52%)	21 (84%)	0.032
Distended GB	23 (92%)	21 (84%)	0.667
Murphy's sign	14 (56%)	14 (56%)	0.999
Pericholecystic fluid	6 (24%)	5 (20%)	0.999
Tense distended GB	18 (72%)	12 (48%)	0.085
Turbid bile/pus	4 (16%)	1 (4%)	0.349
Severe adhesions	3 (12%)	11 (44%)	0.059
GB decompression	16 (64%)	3 (12%)	0
Use of Retrieval bag	4 (16%)	1 (4%)	0.349
Subhepatic drain	7 (28%)	2 (8%)	0.138
Use of 5th port	1 (4%)	0	0.999
Epigastric port enlargement	2 (8%)	2 (8%)	0.999
Operation time	69.4 ± 29.59	66.4 ± 15.97	0.8004
Blood loss	159.6 ± 58.11	146.8 ± 52.69	0.418
Total hospital stay	4.16 ± 1.21	8.6 ± 2.04	0.0001
Conversion rate	16%	8%	0.667
Postoperative complications	24%	8%	0.084
